# Current and Emerging Approaches for Studying Inter-Organelle Membrane Contact Sites

**DOI:** 10.3389/fcell.2020.00195

**Published:** 2020-03-27

**Authors:** Xue Huang, Chen Jiang, Lihua Yu, Aimin Yang

**Affiliations:** School of Life Sciences, Chongqing University, Chongqing, China

**Keywords:** membrane contact sites, electron microscopy, super-resolution microscopy, FRET, proximity ligation assay, bimolecular fluorescence complementation, BioID, APEX

## Abstract

Inter-organelle membrane contact sites (MCSs) are classically defined as areas of close proximity between heterologous membranes and established by specific proteins (termed tethers). The interest on MCSs has rapidly increased in the last years, since MCSs play a crucial role in the transfer of cellular components between different organelles and have been involved in important cellular functions such as apoptosis, organelle division and biogenesis, and cell growth. Recently, an unprecedented depth and breadth in insights into the details of MCSs have been uncovered. On one hand, extensive MCSs (organelles interactome) are revealed by comprehensive analysis of organelle network with high temporal-spatial resolution at the system level. On the other hand, more and more tethers involving in MCSs are identified and further works are focusing on addressing the role of these tethers in regulating the function of MCSs at the molecular level. These enormous progresses largely depend on the powerful approaches, including several different types of microscopies and various biochemical techniques. These approaches have greatly accelerated recent advances in MCSs at the system and molecular level. In this review, we summarize the current and emerging approaches for studying MCSs, such as various microscopies, proximity-driven fluorescent signal generation and proximity-dependent biotinylation. In addition, we highlight the advantages and disadvantages of the techniques to provide a general guidance for the study of MCSs.

## Introduction

A defining characteristic of eukaryotic cells is the presence of membrane-bound organelles surrounded by plasma membrane (PM). Inter-organelle membrane contact sites (MCSs) are classically defined as areas of close proximity between heterologous membranes. At MCSs, specific proteins (termed tethers) hold two organelles together and mediate the transfer of cytoplasmic materials between two organelles (Table 1; Helle et al., 2013; Eisenberg-Bord et al., 2016). The interest on MCSs has rapidly increased in the last few years, since MCSs have been involved in important cellular functions such as apoptosis, cell growth, organelle division and biogenesis (Cohen et al., 2018; Scorrano et al., 2019). Emerging evidence suggests that MCSs play a crucial role in the transfer of cellular components between different organelles, which controls exchange of cellular signaling and regulates organelle membrane dynamics (Friedman et al., 2011; Prinz, 2014; Lahiri et al., 2015; Stefan et al., 2017). For instance, ER-mitochondria MCS is responsible for Ca^2+^ exchange and non-vesicular transfer of phospholipid between mitochondria and ER (Hayashi et al., 2009; Naon and Scorrano, 2014; Krols et al., 2016). The ER-related MCSs, including ER-mitochondria MCS and ER-PM MCS, contribute to autophagosome biogenesis (Hamasaki et al., 2013; Bockler and Westermann, 2014; Garofalo et al., 2016; Nascimbeni et al., 2017). The lysosome-related MCSs in eukaryotic cells act as a dynamic network for nutrient uptake, metabolic control, macromolecule degradation and signaling (Mc Donald and Krainc, 2017; Lawrence and Zoncu, 2019). Therefore, MCSs exist widely in cell and ensure signaling exchange and materials transfer between two different organelles (Table 1).

**TABLE 1:** Summary of membrane contact sites (MCSs).

Contact site	Date Of Discovery	Organism	Proposed function	Used Tools for MCSs	*Identified tethers*
ER-PM	1957 (Porter and Palade, 1957)	Mammalian	lipid transfer; activation of store-operated Ca^2+^ entry; autophagosome biogenesis	EM, ET, Confocal, FM, TIRFM	E-Syts (Schauder et al., 2014), GRAMD2a (Besprozvannaya et al., 2018), VAP-B-Nir2 (Kim et al., 2015), VAP-ORPs (Jansen et al., 2011), JPs (Takeshima et al., 2000; Landstrom et al., 2007), STIM1-Orail-hTRPC1 (Liou et al., 2007; Jardin et al., 2008)
		Yeast	membrane complex formation and localization	EM, FM, Confocal, FRET	Scs2-Scs22 (Manford et al., 2012), Tcb1/2/3 (Manford et al., 2012), Osh2/3 (Levine and Munro, 2001), Ist2 (Manford et al., 2012)
ER-Mitochondria	1959 (Copeland and Dalton, 1959)	Mammalian	Ca^2+^ exchange; lipid exchange; scission of mitochondria; autophagosome biogenesis	EM, FM, Confocal, PLA, FRET, SR-FACT	IP3R-GRP75-VDAC (Rizzuto et al., 1993; Szabadkai et al., 2006; D’Eletto et al., 2018), GRP75-TG2 (D’Eletto et al., 2018), BAP31-FIS1 (Iwasawa et al., 2011), Mfn2-Mfn2/Mfn1-Mfn2 (Naon et al., 2016), VAP-PTPIP51 (De Vos et al., 2012), Lam6 (Elbaz-Alon et al., 2015)
		Yeast (ERMES)	lipid exchange; phospholipid synthesis and cell growth; sterols transport	EM, FM, Confocal, SIM, TIRFM	ERMES complex (Kornmann et al., 2009; Kawano et al., 2018), Gem1 (Kornmann et al., 2011), Mmmr1 (Swayne et al., 2011), TOM5 (Lahiri et al., 2014), Lam6 (Elbaz-Alon et al., 2015), Ltc1-TOM70/71 (Murley et al., 2015), Num1 (Lackner et al., 2013)
ER-Endosome	2009 (Rocha et al., 2009)	Mammalian	sterol sensing and endosome positioning; cholesterol transfer; endosomal tubule fission regulation	EM, FM, Confocal, 3D-SIM, PLA, FRET	VAP-A-STARD3/STARD3NL (Alpy et al., 2013), VAP-A-ORP1L (Rocha et al., 2009), ORP5-NPC1 (Du et al., 2011), Protrudin (Raiborg et al., 2015), PTP1B-EGFR (Eden et al., 2010), Rab5 (Hsu et al., 2018), Spastin-IST1 (Allison et al., 2017), VAP-OSBP-PI4P (Dong et al., 2016), TMCC1-Coronin1C (Hoyer et al., 2018), PTP1B-G-CSFR (Palande et al., 2011)
ER-Golgi	1988 (Schweizer et al., 1988)	Mammalian	lipid exchange	EM, FM, Cryo-EM, Confocal, FRET	VAP-PI4P-OSBP (Mesmin et al., 2013; Jamecna et al., 2019), VAP-CERT (Hanada et al., 2003), VAP-FAPP2 (D’Angelo et al., 2007), VAP-Nir2 (Litvak et al., 2005)
		Yeast	ceramides transfer	EM, FM, APEX2	Nvj2 (Liu et al., 2017)
ER-LD	2006 (Robenek et al., 2006)	Mammalian	LD growth regulation	Confocal, SIM, APEX	Rab18-NRZ (NAG-RINT1-ZW10) (Xu et al., 2018)
		C. elegans	LD expansion	EM, Confocal	FATP1-DGAT2 (Xu et al., 2012)
		Drosophila	LD growth regulation	EM, ET, Confocal, LLSM	Seipin (Wang et al., 2016)
ER-Peroxisome	1987 (Yamamoto and Fahimi, 1987)	Mammalian	peroxisome growth; lipid homeostasis	SIM	VAPs-ACBD4 (Costello et al., 2017), VAPs-ACBD5 (Hua et al., 2017)
		Yeast	peroxisome growth	Confocal	Pex3-Inp1-Pex3 (Knoblach et al., 2013), Pex30 (Joshi et al., 2018)
ER-Lysosome	2018 (Atakpa et al., 2018)	Mammalian	Ca^2+^ exchange	TIRFM, STORM, LLSM, FIB-SEM	unknown
ER-Autophagosome	2016 (Wijdeven et al., 2016)	Mammalian	lipid transfer	Cryo-EM, Confocal, FRET	LTP-ATG2, VAP-A-ORP1L (Wijdeven et al., 2016)
ER-Vacuole	2000 (Pan et al., 2000)	Yeast	selectively sterols transport	FM, BiFC	Ltc1-Vac8 (Murley et al., 2015; Kakimoto et al., 2018), Nvj3-Mdm1 (Henne et al., 2015)
ER-DB	2020 (Dong et al., 2020)	Mammalian	unknown	SR-FACT	unknown
Mitochondria-Golgi	2007 (Ouasti et al., 2007)	Mammalian	apoptosis related	TEM, FM	Fas (CD95/Apo1) (Ouasti et al., 2007)
Mitochondria-Lysosome	2002 (Ponka et al., 2002)	Mammalian	unknown	EM, Confocal, SIM, FRET, SR-FACT	unknown
Mitochondria-Endosome	2002 (Ponka et al., 2002)	Mammalian	early endosomal-mitochondrial contacts and transferrin uptake regulation	EM, FM, SR-FACT	Rab5 (Hsu et al., 2018)
Mitochondria-Peroxisome	2007 (Schrader and Yoon, 2007)	Mammalian	unknown	LLSM, BiFC, Confocal, STEDM	unknown
Mitochondria-LD	2011 (Wang et al., 2011)	Mammalian	contact regulation	EM, SR-FACT	Perilipin-5 (Wang et al., 2011)
Mitochondria-Vacuole (vCLAMP)	2014 (Honscher et al., 2014)	Yeast	survival in starvation and stress	EM, BiFC	TOM40-Vps39, Mcp1-Vps13 (Gonzalez Montoro et al., 2018) (vCLAMP)
Mitochondria-DB	2020 (Dong et al., 2020)	Mammalian	mitochondrial fission	SR-FACT	unknown
Golgi-Autophagosome	2015 (Biazik et al., 2015)	Mammalian	autophagosome formation	EM, ET	unknown
Lysosome-Peroxisome	2015 (Chu et al., 2015)	Mammalian	cholesterol transfer	FM, LLSM	Syt7 (Chu et al., 2015; Valm et al., 2017)
Lysosome-Autophagosome	1992 (Lawrence and Brown, 1992)	Mammalian	autophagosome formation	EM, ET, FM	Rab7-HOPS related complex (Jiang et al., 2014)
		Yeast	autophagosome formation	EM, ET, FM	Ypt7-HOPS related complex (Kirisako et al., 1999)
Lysosome-Endosome	1999 (Ohashi et al., 1999)	Mammalian	proper lysosomal digestive functions	EM, FM	Vamp8-Syntaxin 7 (Mullock et al., 2000),
		Yeast	endosome fusion regulation	FM	Rab7-HOPS-Rab7 (Nordmann et al., 2010; Balderhaar and Ungermann, 2013)
Lysosome-LD	2014 (Dugail, 2014)	Mammalian	lipid homeostasis	EM, FM, LLSM	PLIN2-HSC70-LAMP2A (Olzmann and Carvalho, 2019)
Lysosome-Golgi	2018 (Hao et al., 2018)	Mammalian	amino acid supply response	FM, PLA	mTORC1-Rheb (Hao et al., 2018)
Peroxisome-LD	2006 (Binns et al., 2006)	Mammalian	fatty acid trafficking	FIB-SEM, Confocal, LLSM, BiFC	M1 Spastin-ABCD1-ESCRT-III (Prinz et al., 2020)
		Yeast	fatty acid trafficking	FIB-SEM, Confocal, LLSM, BiFC	M1 Spastin-ABCD1-ESCRT-III (Prinz et al., 2020)
Peroxisome-PM	2018 (Kakimoto et al., 2018)	Yeast	unknown	BiFC	unknown
Peroxisome-Vacuole	2018 (Kakimoto et al., 2018)	Yeast	unknown	BiFC	unknown
Nucleus-Vacuole	1976 (Severs et al., 1976)	Yeast	microautophagy related	BiFC	Nvj1p-Vac8p (Kvam and Goldfarb, 2006), Lam6-Vac8 (Elbaz-Alon et al., 2015)
Nucleus-DB	2020 (Dong et al., 2020)	Mammalian	nuclear membrane formation	SR-FACT	unknown
LD-PM	2018 (Kakimoto et al., 2018)	Yeast	unknown	BiFC	unknown
LD-Vacuole	2018 (Kakimoto et al., 2018)	Yeast	unknown	BiFC	unknown
Vacuole-PM	2018 (Kakimoto et al., 2018)	Yeast	unknown	BiFC	unknown

*The table summarizes MCSs and related information in different organisms. PM, plasma membrane; LD, lipid droplet; DB, Dark-vacuole body; vCLAMP, vacuolar and mitochondrial patch; ERMES, ER-mitochondria encounter structure; EM, electron microscopy; TEM, transmission electron microscope; Cryo-EM, cryogenic electron microscopy; ET, electron tomography; FIB-SEM, focused ion beam-scanning EM; FM, fluorescence microscope; Confocal, confocal microscopy; SIM, structured illumination microscopy; 3D-SIM, three-dimensional structured illumination microscopy; TIRFM, total internal reflection fluorescence microscopy; SR-FACT, super-resolution fluorescence-assisted diffraction computational tomography; LLSM, lattice light-sheet microscopy; STORM, stochastic optical reconstruction microscopy; STEDM, stimulated emission depletion microscopy; PLA, proximity ligation assay; FRET, fluorescence resonance energy transfer; BiFC, bimolecular fluorescence complementation; APEX, engineered peroxidase; APEX2, engineered variant of soybean ascorbate peroxidase.*

The dysfunction of MCSs has been implicated in neurodegenerative disorders and cancer (Prinz et al., 2020). To date, most investigations into the roles of MCSs dysfunction in disease have focused on ER-mitochondria MCSs (Paillusson et al., 2016). The mutation of the tether Mnd, VAPB or REEP defects ER-mitochondria MCS and Ca^2+^ signaling transfer, and finally leads to neurodegenerative disease (Vance et al., 1997; Nishimura et al., 2004; Schon and Area-Gomez, 2013; Stoica et al., 2014; Bernard-Marissal et al., 2015; Lim et al., 2015). In addition, the mutation of the tether BAP31 dysregulates ER-Golgi crosstalk and impacts Golgi apparatus, and thereby causing the X-link phenotype with deafness, dystonia and central hypomyelination (Cacciagli et al., 2013).

**FIGURE 1:**
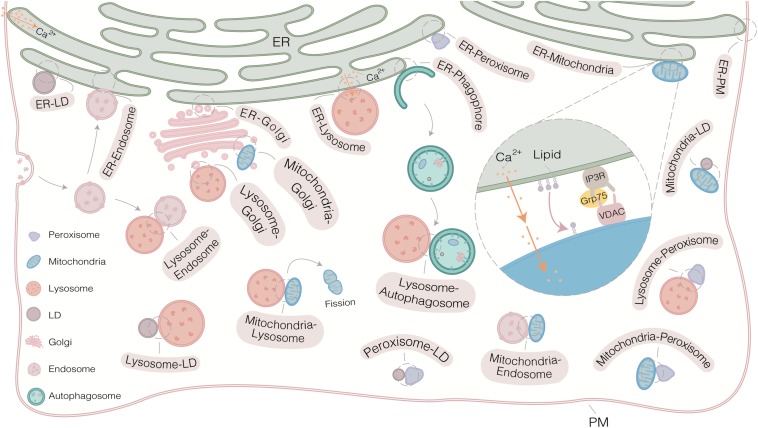
Extensive membrane contact sites (MCSs) in mammalian cells. MCSs exist widely in cell and regulate exchanges signaling and regulates cellular functions. The common strategy for studying MCSs consists of various microscopies and biochemical approaches. Multiple canonical protein complexes have been reported to regulate MCSs, such as VDAC-IP3R-Grp 75 complex at ER-mitochondria MCS. Some MCSs, such as mitochondria-endosome MCS, Golgi-autophagosome MCS and lysosome-peroxisome MCS, are not shown due to limited space in this figure.

**FIGURE 2:**
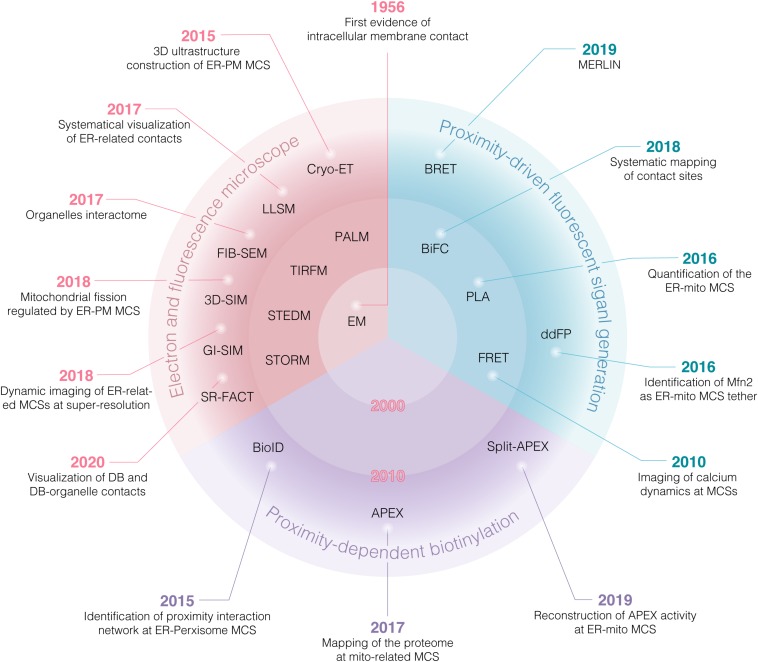
Overview of various approaches for studying MCSs. Various approaches have greatly accelerated recent advances in MCSs at the system and molecular level. Before 2000, EM and confocal microscopy were most accessible tools. EM provided first evidence of intracellular membrane contact at nanoscale resolution, while confocal microscopy has been employed to reconstruct the 3D imaging of ER-mitochondria juxtaposition as sites of Ca^2+^ transfer between both organelles. Since 2010, super resolution microscopy (SRM) was increasingly used to visualize MCSs. Furthermore, proximity-driven fluorescent signal generation approaches, such as FRET, PLA, BiFC and ddFP were exploited to identify the potential MCSs. Notably, by combination with proteomic analysis, proximity-dependent biotinylation approaches mediated by BioID and APEX provide a promising strategy for global mapping MCSs and identification of tethers involving in MCSs, termed as “tether-omics.” BRET, bioluminescence resonance energy transfer; BioID, proximity-dependent biotin identification; APEX, ascorbate peroxidase; MERLIN, mitochondria-ER length indicator nanosensor; FIB-SEM, focused ion beam-scanning EM; PALM, photoactivation localization microscopy; LLSM, lattice light-sheet microscopy; SR-FACT, super-resolution fluorescence-assisted diffraction computational tomography.

Electron microscopy (EM) provides the first evidence for the existence of sites of physical interaction between ER and mitochondria in the 1950s (Bernhard and Rouiller, 1956; Copeland and Dalton, 1959). Since then, however, the research on MCSs has been proceeded slowly due to lack of suitable study tools. From the early of 21st century, an unprecedented depth and breadth in insights into the details of MCSs have been uncovered gradually. On one hand, extensive MCSs (organelles interactome) are revealed by comprehensive analysis of organelle network with high temporal-spatial resolution at the system level (Figure 1). On the other hand, more and more tethers involving in MCSs are identified and further works are focusing on addressing the role of these tethers in regulating the function of MCSs at the molecular level (Table 1; Jansen et al., 2011; Elbaz-Alon et al., 2015; Jing et al., 2015; Kim et al., 2015; Besprozvannaya et al., 2018). These enormous progresses largely depend on the powerful approaches, including several different types of microscopies and various biochemical techniques (Figure 1). These approaches have greatly accelerated recent advances in MCSs at the molecular and system level (Figure 2). In this review, the current and emerging approaches for studying MCSs are summarized. In addition, the advantages and disadvantages of the approaches are highlighted to provide a general guidance for tool selection and optimization for the study of MCSs.

## An Overview of the Approaches for Studying MCSs

Visualization is a prerequisite for study of MCSs. The “membrane contact” was originated from an observation that topographical proximity between mitochondria and ER in cells of the pseudobranch gland of a teleost by EM (Bernhard and Rouiller, 1956; Copeland and Dalton, 1959). EM of cell specimen at the nanometer scale make observation of subcellular structures and MCSs possible. Recently, EM and its variants, such as focused ion beam-scanning EM (FIB-SEM) and electron tomography (ET), are widely employed to observe MCSs. High resolution of three-dimensional (3D) structure at ER-PM MCS in COS-7 cells was obtained by cryo-ET (Fernandez-Busnadiego et al., 2015). Meanwhile, various light microscopies based on fluorescence were developed in living cells. Confocal microscopy is used to visualize subcellular localization of fluorescent fusion membrane proteins and has been employed to reconstruct 3D imaging of ER-mitochondria juxtaposition as sites of Ca^2+^ transfer between both organelles in HeLa cells (Rizzuto et al., 1998). Lattice light-sheet microscopy (LLSM) was developed by using ultrathin light sheets from two-dimensional optical lattices to reveal organelle interactome at the systems-level in COS-7, HEK293 and MEF cells (Valm et al., 2017; D’Eletto et al., 2018). Super-resolution fluorescence microscopy (SRM) offers a unique window with extreme high temporal and spatial resolution for MCSs (Sydor et al., 2015; Sezgin, 2017; Jing et al., 2019). Recently, grazing incidence structured illumination microscopy (GI-SIM), one of SRM, was developed and applied to visualize ER-mitochondria MCS in COS-7 and U2OS cells (Guo Y. et al., 2018). In brief, various microscopies provide a large of direct evidence for visualization of MCSs and greatly facilitate the development of the field.

In combination of confocal microscopy, a variety of biochemical techniques, including proximity ligation assay (PLA) (Soderberg et al., 2006), fluorescence resonance energy transfer (FRET) (Csordas et al., 2010), bimolecular fluorescence complementation (BiFC) (Cabantous et al., 2005; Magliery et al., 2005) and dimerization-dependent fluorescent proteins (ddFP) (Alford et al., 2012a) were designed to observe and identify MCSs. These biochemical techniques are dependent on proximity-driven signal generation and amplification. Briefly, two fragments or proteins, fused with tethers or membrane proteins of different organelles, are each no signal or low signal on their own but are reconstituted to give strong signal when driven together by close membrane-membrane proximity. These techniques are widely employed to visualize MCSs and identify tethers involving in MCSs. In recent years, many studies focused on identification of new tethers. To achieve this purpose, two novel tools, BioID and APEX based on proximity-driven biotinylation were engineered and applied to identify new tethers and even systematically map MCSs in COS-7 and HEK293 cells (Hua et al., 2015; Cho et al., 2017).

Overall, these approaches have greatly accelerated research progress in MCSs (Table 1 and Figure 2). It is expected that new approaches, such as SRM and proximity-driven biotinylation coupled with mass spectrometry (MS)-based proteomics, will facilitate the research of MCSs at the molecular and system level.

## Visualization of MCSs by EM and SRM

Electron microscopy provides the first evidence for ER-mitochondria MCS that improves the transfer of Ca^2+^ signal between both organelles in cells of the pseudobranch gland of a teleost (Copeland and Dalton, 1959; Rizzuto et al., 1998). Recently, EM and its variants, such as electron tomography (ET) and focused ion beam-scanning EM (FIB-SEM), are widely used to observe MCSs. High resolution structure of ER-mitochondria MCS was obtained by ET, an extension of traditional transmission EM (Csordas et al., 2006; de Brito and Scorrano, 2008). Two well-known structures, ER-mitochondria encounter structure (ERMES) and vacuolar and mitochondrial patch (vCLAMP) in yeast were directly observed by EM and ET (Kornmann et al., 2009; Honscher et al., 2014). The 3D ultrastructure of ER-PM MCS in COS-7 cells was visualized in close-to-native conditions by cryo-ET (Fernandez-Busnadiego et al., 2015; Fernandez-Busnadiego, 2016; Collado and Fernandez-Busnadiego, 2017). In FIB-SEM, a highly focused gallium ion beam ablates a thin layer of the sample after which the newly exposed surface is imaged with the scanning electron beam (Drobne, 2013; Fermie et al., 2018). FIB-SEM has recently been employed to systematically visualize ER MCSs with multiple other membranes including mitochondria and PM in neurons of mice (Wu et al., 2017). EM is regarded as the “gold standard” and exquisitely suited for investigation of MCSs, which provides fine architecture and spatial resolution of subcellular compartments. To some extent, EM has some drawbacks, such as fixation procedures, time-consuming and low through-put, restricting EM utility in living cells.

To enable visualization of MCSs in living cells, genetically encoded fluorescent proteins are tagged to resident proteins or tethers of different membranes so that MCSs become visible under multispectral fluorescence microscopy. Confocal microscopy is one of the most common methods to visualize subcellular localization of fluorescent membrane proteins. It has been employed to deconvolute and reconstruct 3D imaging of ER-mitochondria juxtaposition in living HeLa cells (Rizzuto et al., 1998) and of ERMES in yeast (Kornmann et al., 2009). To expand observation of dynamic multiple MCSs, lattice light-sheet microscopy (LLSM) was developed by using ultrathin light sheets from two-dimensional optical lattices (Chen et al., 2014). Multispectral imaging and computational analysis were introduced to visualize and quantify organelle interactome between six different organelles in COS-7 cells, such as ER, Golgi, lysosome, peroxisome, mitochondria and lipid droplet (LD) (Valm et al., 2017).

The dynamics and fine structure of MCSs require research tool with extreme high temporal and spatial resolution. Super-resolution fluorescence microscopy (SRM) offers a unique window for the study of MCSs. Several SRMs, such as stimulated emission depletion microscopy (STEDM), photoactivation localization microscopy (PALM) and stochastic optical reconstruction microscopy (STORM), structured illumination microscopy (SIM), have been developed for imaging of nanoscale structural details and dynamics of MCSs, such as ER-PM MCS (Hsieh et al., 2017; Nascimbeni et al., 2017), ER-mitochondria MCS (Shim et al., 2012; Modi et al., 2019), ER-lysosome MCS (Shim et al., 2012), mitochondria-peroxisome MCS (Galiani et al., 2016). Among these SRMs, SIM is suitable for fast live-cell imaging and has been used to reveal numerous subcellular structures and dynamics (Li et al., 2015; Nixon-Abell et al., 2016; Zhanghao et al., 2019). Recently, ER-PM MCS in U2OS, Jurkat T and HEK293 cells was observed by SIM with total internal reflection fluorescence microscopy (TIRF-SIM) (Guo M. et al., 2018; Kang et al., 2019). The grazing incidence SIM (GI-SIM) was developed and applied to visualize ER-mitochondria, ER-late endosome and ER-lysosome MCSs in COS-7 and U2OS cells (Guo Y. et al., 2018). In addition, SIM based on Hessian matrixes excels in extending the spatiotemporal resolution in live cells and is expected to apply for studying MCSs in COS-7 cells (Huang et al., 2018). More importantly, super-resolution fluorescence-assisted diffraction computational tomography (SR-FACT) was developed by combination of label-free three-dimensional optical diffraction tomography (ODT) with two-dimensional fluorescence Hessian structured illumination microscopy (Dong et al., 2020). The SR-FACT enable label-free visualization of various subcellular structures and complete division process of a COS-7 cell. By using SR-FACT, novel subcellular structures named dark-vacuole bodies (DB) were observed, and intensively contact with organelles such as mitochondria and nuclear membrane in COS-7 cells (Dong et al., 2020).

## Identification of MCSs by Proximity-Driven Fluorescent Signal Generation

**FIGURE 3:**
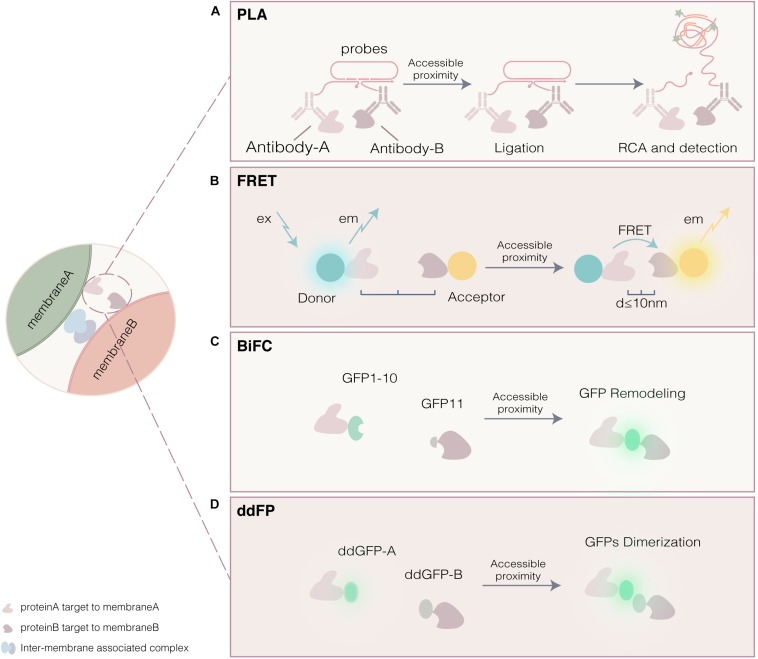
Proximity-driven fluorescent signal generation approaches. **(A)** Proximity ligation assay (PLA). Single-stranded oligonucleotides are conjugated to antibody of proteins. Membrane proximity serves to template the hybridization of circularization oligonucleotides, which is then joined by ligation into a circular DNA molecule and followed by amplification of the signal by rolling-circle amplification (RCA). **(B)** Fluorescence resonance energy transfer (FRET). The photon energy transfers from excited donor to acceptor since the accessible proximity. **(C)** Bimolecular fluorescence complementation (BiFC). Accessible proximity made GFP1-10 and GFP11 remodel into a mature fluorescent protein. **(D)** Dimerization-dependent fluorescent proteins (ddFP). Two GFP monomer (ddGFP-A and ddGFP-B) with weak signal form a dimer with fluorescent strong signal since the accessible proximity.

Confocal microscopy is used to visualize subcellular localization of fluorescent membrane proteins, rather than suitable to study MCSs. On one hand, co-localization of resident proteins or tethers of different membranes observed by confocal microscopy, however, doesn’t mean the existence of MCS. On the other hand, because of continuing organelles movement, dynamic MCSs are difficult to detect by confocal microscopy alone with low resolution. The proximity-driven fluorescent signal generation enable identification of MCSs with more reliability and easy operation. Briefly, two fragments or proteins fused with organelle markers or tethers, reside on outer membrane of different organelles, are each no signal or low signal on their own but are reconstituted to give strong fluorescent signal when driven together by membrane proximity. These approaches are widely used to visualize MCSs and identify important tethers involving in MCSs (Figure 3 and Table 1).

### PLA

Proximity ligation assay (PLA) is a unique method for protein detection. In PLA, single-stranded oligonucleotides are conjugated to affinity binders or antibody of proteins, followed by amplification of the signal by rolling-circle amplification (RCA) and detection of complementary fluorophore labeled oligonucleotides (Fredriksson et al., 2002; Schallmeiner et al., 2007; Soderberg et al., 2008). PLA has success to observe interaction of individual endogenous protein complexes *in situ* (Soderberg et al., 2006; Fredriksson et al., 2007), which validates protein-protein interactions in multiplexed proteins.

Recently, PLA has been employed to address MCSs. Dual binding by a pair of proximity probes (antibodies with attached DNA strands) to two membrane resident proteins or tethers serves to template the hybridization of circularization oligonucleotides, which is then joined by ligation into a circular DNA molecule (Figure 3A). ER-mitochondria MCS was visualized and quantified by using the close proximity between voltage-dependent anion channel 1 (VDAC1, localizes in outer mitochondrial membrane) and inositol 1,4,5-triphosphate receptor (IP3R, localizes in ER membrane) at the mitochondria-associated membranes (MAMs) interface in human HuH7 cells (Tubbs and Rieusset, 2016). The VDAC1-IP3R PLA system was used to reveal the role of ER-mitochondria tethering complex VAPB-PTPIP51 in regulating autophagy in HeLa and HEK293 cells (Gomez-Suaga et al., 2017). In addition, by using VDAC1-IP3R PLA system, Stoica et al. found that ALS/FTD-associated FUS activates GSK-3β to disrupt the VAPB-PTPIP51 interaction and ER-mitochondria associations in NSC-34 mouse motor neuron cells (Stoica et al., 2016), and Thomas, et al. revealed that phenformin blocks MAMs that support autophagy in HEK293 cells (Thomas et al., 2018). ER-lysosome MCS was detected by PLA, and further experiment confirmed that the clusters of IP3R populate ER-lysosome MCS and facilitate local delivery of Ca^2+^ from the ER to lysosome (Atakpa et al., 2018).

### FRET

Fluorescence resonance energy transfer is one of most accessible technologies that allow for detecting protein-protein interaction at super resolution. FRET was primally from Förster’s theory, in which dipole-dipole interaction made the photon energy transfer from excited donor to acceptor between an energy donor-acceptor fluorescent pair when the distance between donor and acceptor was 1 to 10 nm. FRET is a non-destructive method of spectroscopy and therefore applied to observe the signal of MCSs in living cells (Zimmer, 2002; Pietraszewska-Bogiel and Gadella, 2011; Marx, 2017; Figure 3B). Fluorescent proteins were derived from 1980s when green fluorescent protein (GFP) of *Aequorea victoria* was exploited as a GFP-chimera targeted to specific organelles membrane and further optimized to the mutants, such as RFP and BFP (Rizzuto et al., 1996; Zimmer, 2002). Normally, fluorescent proteins fused with membrane proteins or tethers are used as donor/acceptor probes. The combination between fluorescent proteins and FRET brings a great progress for the great integration with targeted protein. FRET has been exploited to be a useful tool by combination with other technologies. Combination between fluorescence lifetime imaging (FLIM) and FRET offers direct evidence of temporal membrane proximity at high resolution (Bastiaens and Squire, 1999; Elangovan et al., 2002; Sekar and Periasamy, 2003). Photoswitching FRET (psFRET), a revised version of photobleaching FRET (pb FRET) of which detection of fluorescence signal required only imaging of donor before and after photobleaching of acceptor (Wouters et al., 1998). Improvement of psFRET make photobleach to be switched “off” and be re-detectable (Rainey and Patterson, 2019). Recent work systematically assessed the FRET from principle to screening of donor-acceptor pair (Algar et al., 2019).

Fluorescence resonance energy transfer is an indispensable experimental tool for the study of the MCSs (Jing et al., 2015; Subedi et al., 2018). Tandem GFP pairs allowed for detecting the alteration of intracellular Ca^2+^ level at mitochondria-ER MCS in HeLa and HEK293 cells, in which tandem expression of BFP-CBD (26-residue containing calmodulin-binding domain)-EGFP was sensitive to change of Ca^2+^ flux that causes a structural alteration of CBD and a destroyed FRET pair (Miyawaki et al., 1997; Romoser et al., 1997). Cooperation between FRET and total internal reflection microscopy (TIRFM) was designed to study ER-PM MCS in RBL-2H3 (mice) and HeLa cells (Poteser et al., 2016; Chen et al., 2017; Chang et al., 2018). FRET was applied to study lipid transfer regulated by oxysterol-binding protein (OSBP) at the MCSs between ER and other organelles in human RPE1 cells (Jamecna et al., 2019). Furthermore, another improved method is that FK506-binding protein 12 (FKBP 12) and FKBP 12-rapamycin binding domain (FRB) were anchored to different organelles by each resident membrane protein, respectively. As rapamycin induction, the closely spatial FKBP and FRB interact with each other, which activates FRET signal (Inoue et al., 2005). This method was applied to study ER-mitochondria MCS in rat H9C2 cardiomyoblast cells and basophilic leukemia (RBL)-2H3 cells (Csordas et al., 2010) and identify ER-mitochondria MCS tether Mfn2 in MEF cells (Naon et al., 2016). In another study, FRET venus-mTurquoise2 pair was fused to TOM20 and LAMP1 respectively, to study the regulation of mitochondria-lysosome MCS mediated by mitochondria fission via Rab7 GTP hydrolysis in HeLa cells (Wong et al., 2018).

A variant of FRET technique, bioluminescence resonance energy transfer (BRET) has been developed to study protein-protein interactions (Pfleger and Eidne, 2006). In BRET, the donor is luciferase enzyme which catalyzes a bioluminescent oxidation, and then the energy is transferred to the acceptor by resonance if the protein-protein interacts occurs. Compare with FRET, BRET does not require sample illumination to excite the donor and has been emerged as a powerful tool for the study of protein-protein interactions (Perroy et al., 2004; De, 2011; Mo and Fu, 2016). Recently, a novel BRET-based biosensor with Renilla Luciferase 8 (RLuc) acting as a donor and mVenus as an acceptor, named MERLIN (mitochondria-ER length indicator nanosensor), was presented for the analysis of distances between ER and mitochondria in COS1 and HCT116 cells (Hertlein et al., 2020). In MERLIN, mVenus was targeted to mitochondria by the alpha-helical C-terminal domain of Bcl-xL (B33C) and RLuc was targeted to ER by a truncated non-functional variant of calnexin (sCal). The further experiments have demonstrated that MERLIN is a powerful and innovative tool for the investigation of ER-mitochondria MCS.

### Bimolecular Fluorescence Complementation

Bimolecular complementation (BiC) system was successful applied in multiple proteins, such as ubiquitin (Johnsson and Varshavsky, 1994), β-lactamase (Galarneau et al., 2002), firefly (Luker et al., 2004) and fluorescent proteins. Bimolecular fluorescence complementation has emerged as a key technique to visualize protein-protein interactions in living cells. In BiFC system, split-fluorescent protein composes of two complementary protein residues, each no fluorescent signal on their own but are reassemble to give the bright fluorescence when driven together by protein interaction. In the earlier split-GFP system, GFP protein was divided into GFP-N (residues 1–157) and GFP-C (residues 158–230) with fused to leucine zipper, respectively. Leucine zipper interaction drive refold of GFP protein and the green fluorescence was recovered (Magliery et al., 2005). Because of low efficiency of fluorescence recovery, a more effective system, split super-folder GFP was engineered for efficient self-complementation without leucine zipper or other protein-protein interaction (Cabantous et al., 2005). In this method, GFP protein was divided into GFP1-10 (residues 1–214) fragment and GFP11 (residues 214–230) fragment, of which GFP 1-10 fragment contains three residues of the fluorophore. Only if GFP1-10 complement with the conserved residue E222 at GFP11, the fluorophore is reactive with brightest green fluorescence (Cabantous et al., 2005). For successful construction of split-fluorescent system, each individual protein residue cannot show any protein activity, and each individual protein residue cannot show a distinct fluorescent activity, instead a strong signal should be detected when reassembled (Magliery et al., 2005; Shekhawat and Ghosh, 2011). Notably, engineered GFP mutants were always considered due to the limited GFP fluorescent intensity (EGFP and Venus) (Wiens and Campbell, 2018). For the signal irreversibility, this method was commonly competent to detect transient protein-protein interaction (Magliery et al., 2005), instead of temporal information of protein-protein interaction.

Bimolecular fluorescence complementation is able to sensitively detect MCSs, where the contact signal always shows stable dots (Figure 3C). ER membrane protein Ifa38 and mitochondrial outer membrane protein TOM71 fused to GFP 1-10 and GFP11, respectively, highlight the signal of ER-mitochondria MCS as dots in yeast and HeLa cell. LD-peroxisome MCS can also be labeled with this split system mediated by LD protein Erg6 and peroxisome protein Pex3 in yeast (Kakimoto et al., 2018). The GFP variants, such as Venus, mCherry, and FusionRed, were also exploited to study MCSs (Toulmay and Prinz, 2012; Lahiri et al., 2014; Wiens and Campbell, 2018). Venus protein was derived from GFP and carries five amino acids mutation with improved brightness (Rekas et al., 2002). Similar with split GFP, split-Venus was developed and applied to detect MCSs such as ERMES, ER-LD MCS and vCLAMP in yeast (Shai et al., 2018). More importantly, split-Venus was employed to uncover new MCSs in yeast, such as vCOuPLE (vacuole-plasma membrane contact), pCLIP (plasma membrane-lipid droplet), PerPECs (peroxisome-plasma membrane) and PerVale (peroxisome-vacuole) (Shai et al., 2018). The split-Venus also was used to detect ER-mitochondria junctions, further experiment has demonstrated that ER membrane protein complex (EMC) tethers ER to mitochondria, which is required for phospholipid synthesis and cell growth in yeast (Lahiri et al., 2014). BiFC was employed to detect plastic remodeling of ER-mitochondria MCS and it is demonstrated that ER-mitochondria MCS is dynamic structure that undergoes active remodeling under different cellular needs in human osteosarcoma U2OS cells (Yang et al., 2018).

Recently, to expand BiFC toolset, direct engineering of self-complementing split fluorescent protein was developed by insertion a 32 amino acid spacer between the tenth and eleventh β-strands of GFP. The dual-color endogenous protein was tagged with sfCherry2 11 and GFP 11, revealing that the abundance of ER translocon complex Sec61B reduced in certain peripheral tubules. The new BiFC system offers multiple colors for imaging MCSs in HEK293T cells (Feng et al., 2017). In addition, a split GFP-based contact site sensor (SPLICS) was designed to detect the wide or narrow membrane contacts (narrow: 8–10 nm and wide: 40–50 nm) by the flexible spacer linked between GFP11 and ER targeting sequence. The ER-mitochondria MCS was allowed for detection and monitored by using this sensor in HeLa and HEK293 cells (Cieri et al., 2018).

### ddFP

Dimerization-dependent fluorescent protein (ddFP) is a class of genetically encoded reporters based on the reversible binding of two dark fluorescent protein monomers to form a fluorescent heterodimeric complex, which can be used for detection of protein-protein interactions in living cells (Ding et al., 2015; Mitchell et al., 2018). The yellow or red fluorescent proteins are obligate tetrameric, however, is limited by its tetramerization disruption. To solve this problem, multiple monomeric fluorescence proteins were obtained through amino acid mutation, such as dTomato, mCherry and mStrawberry (Shaner et al., 2004). These monomeric proteins act as basic components to employ the ddFP tool. For earliest version of ddFP construction, a monomeric fluorescent dTomato variant (H162K and A164R) and a suitable “aptamer” (engineered another dTomato monomer) were screened. The “aptamer” allowed to constitute a heterodimer with the dTomato variant. Both two engineered dTomato monomer exhibited a weak red fluorescence, however, the spatial proximity induced formation of the heterodimer through non-covalent interaction to exhibit a brighter fluorescent signal (Alford et al., 2012a). Furthermore, ddGFP was engineered with brighter fluorescent signal exhibited ∼60-fold increase in emission intensity upon heterodimerization. The protein-protein interaction can act as an indicator when two monomers are spatial proximity and thus an ideal tool for the study of MCSs. The advantage of ddFP is its reversibility, which is suitable to measure the dynamics of MCSs (Figure 3D).

By using ddGFP tool, a highly effective indicator of membrane proximity was generated to image MAM interface of ER and mitochondria by fusing two monomers of fluorescent protein to endoplasmic reticulum membrane (ERM) and outer mitochondrial membrane (OMM), respectively in HeLa cells (Alford et al., 2012b). When their spatial distance was less than 20 nm, a heterodimer was reconstructed by non-covalent interaction between monomers and then produce a stronger fluorescent signal. The ddGFP signal at ER-mitochondria MCS reduced when Mfn2, a crucial ER-mitochondria MCS tether, was deleted in MEF cells (Naon et al., 2016). The mutant TDP-43 did not impair mitochondrial bioenergetics by using ddGFP targeted to mitochondria and ER (TOM20-ddGFP and calN-ddGFP, respectively) in HeLa cells (Kawamata et al., 2017).

## Global Mapping MCSs by Proximity-Dependent Biotinylation

**FIGURE 4:**
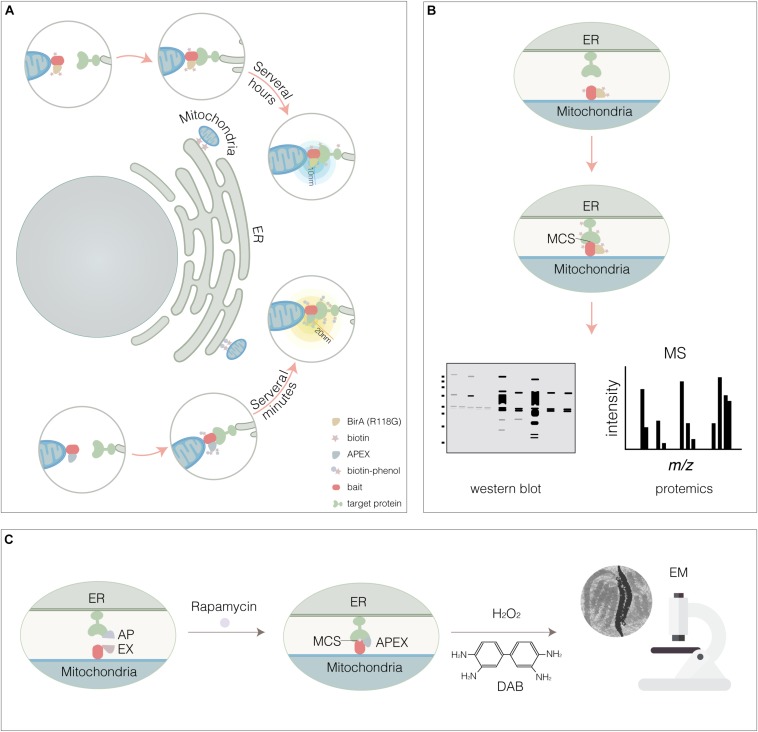
Proximity-dependent biotinylation approaches. **(A)** BioID- or APEX-based proximity labeling. BirA* (Upper panel) or APEX (Lower panel) were fused to mitochondria membrane protein (bait). The potential target that be thought to interact with bait within the accessible proximity (10 nm for BirA*, 20 nm for APEX) would be biotinylated. For BirA*, endogenous biotin was accessible, instead APEX required the exogenous biotin-phenol and H_2_O_2_. **(B)** Cell expressing BirA*-bait or APEX-bait were lysed, then the biotinylated targets were captured by streptavidin-beads and identified by WESTERN BLOT and MS. **(C)** Reconstitution of split-APEX at ER- mitochondria MCS. By AP fused to TOM20^1– 34^ for targeting OMM and EX fused to Cb5^100– 134^ for targeting ERM, respectively, the split APEX enzyme activity was reconstituted and ER-mitochondria MCS was visualized at higher resolution by EM through the combination of the DAB staining.

Inter-organelle membrane contact sites are established and maintained by tethers (Table 1). Although MCSs have been widely observed by EM and fluorescence techniques, tethers remained to be explored. Almost identified MCSs are mediated by multiple tethering proteins or protein complex, rather than only one tether. Some tethers were identified to mediate MCSs by traditional biochemical approaches, such as cell fractionation and pull-down. However, several considerable defects limit their application. Firstly, these approaches fail to catch the transient membrane contact; Secondly, the MCSs might be destroyed when detergent is added; Lastly, the dynamics of MCSs can’t be monitored. The proximity biotinylation approaches such as proximity-based biotin identification (BioID) and ascorbate peroxidase (APEX) tagging are used to label neighboring proteins by generating a reactive biotin derivative (Figure 4). These proximity-dependent biotinylation approaches combined with proteomic analysis provide a promising strategy for global mapping MCSs.

### BioID

Proximity-based biotin identification technology is dependent on BirA, a 35 kD DNA-binding biotin protein ligase that regulates the biotinylation of a subunit of acetyl-CoA carboxylase and inhibits biotin biosynthetic operon (Lane et al., 1964). For biotinylation, native biotin binds with ATP to constitute a biotinoyl-5′-AMP (BioAMP) prior to target to active site of BirA, biotin is then ligated to lysine residue when BAT sequence, a biotin acceptor tag, is recognized by BirA. Compared with BirA, BirA^∗^ (a R118G BirA mutant) with a lower affinity toward BioAMP causes promiscuous protein biotinylation. BioID enables to biotinylate potential proteins in proximity-dependent manner (Cronan, 2005; Gupta et al., 2015; Gingras et al., 2019). Cells expressing a bait resident protein fused to BirA^∗^ tag are incubated with biotin for several hours and then the biotinylated proteins are captured on a streptavidin affinity matrix for identification by LC-MS/MS (Figures 4A,B). BioID technology has proven to be a powerful method for identifying proximal proteins in cells.

The tethers involving in ER-peroxisome MCS were identified by BioID (Hua et al., 2017). PEX16 a key peroxisomal biogenesis protein and initially targets to the ER before the traffic to peroxisomes in COS-7 cells (Hua et al., 2015). By using BioID, a proximity interaction network (70 high-confidence proximal interactors) for PEX16 was mapped in human 293 T-REx Flp-In cells. The further experiment highlighted that ER-resident VAMP-associated proteins A and B (VAPA and VAPB) interact with peroxisomal membrane protein acyl-CoA binding domain containing 5 (ACBD5), which is required to tether two organelles together and thereby facilitates lipid exchange between the organelles in COS-7 cells. BioID was also used to investigate the role of tethers in ER-PM MCS. Unfolded protein response (UPR) PERK-BirA proximity interactome was obtained, and furthermore, actin-binding protein filamin A (FLNA) was identified as a key PERK interactor. The work revealed the role of PERK as a multimodal organizer of MCSs between ER and other vital organelles. As an apical sensor of ER-Ca^2+^ store alterations, PERK was able to tightly couple store depletion to facilitate the expansion of ER-PM MCS through interaction with FLNA and spatial organization of actin cytoskeleton in HEK293T cells (van Vliet et al., 2017).

In addition, as its effectiveness, split-BioID was exploited to uncover the PP1-interacting proteins (PIPs) targeted by protein phosphatase PP1 in HEK293T cells (De Munter et al., 2017). In split-BioID, BirA^∗^ was divided into BirA^∗^-N and BirA^∗^-C (at amino acid 140/141). The enzyme activity of BirA^∗^ is reconstructive after heterodimerization of BirA^∗^-N and BirA^∗^-C fragments.

### APEX

Ascorbate peroxidase is an engineered ascorbate peroxidase. In the presence of hydrogen peroxide, APEX not only catalyzes 3,3′-diaminobenzidine (DAB) to generate an electron-dense product for visualization by EM (Figure 4C; Martell et al., 2012), but also converts a phenolic substrate (biotin-phenol) into a highly reactive radical and covalently tags proximal endogenous proteins (Rhee et al., 2013; Hung et al., 2014). Because the limited sensitivity of APEX precludes applications requiring low APEX expression, more active form APEX2 for intracellular specific protein imaging by EM and spatially-resolved proteomic mapping was obtained by yeast display evolution (Lam et al., 2015). In the presence of the APEX2 substrate biotin-phenol, a brief pulse of hydrogen peroxide (H_2_O_2_, < 1 min) results in the APEX2-catalyzed generation of short-lived, membrane impermeable biotin-phenoxyl radicals that form covalent adducts with electron-rich amino acids in proteins located within a 10–20 nm radius, which enables to mark the potential proteins involving in MCSs (Figures 4A,B).

Ascorbate peroxidase has been successfully applied to localize mitochondria at proteomic level without mitochondria purification (Rhee et al., 2013), and also give insights into the composition and dynamics of LD proteomes in human osteosarcoma U2OS cells (Bersuker et al., 2018). The proteome at ER-mitochondria MCS was mapped by using APEX. The tethers localizing at the MCS between mitochondria and other organelles were identified and validated by combining biochemical subcellular fractionation. For instance, atlastin (ATL2) and reticulon (RTN1 and RTN3) are critical in forming ER-mitochondria MCS in HEK293 cells (Cho et al., 2017). Another work focused on ER-mitochondria MCS using APEX2 (Hung et al., 2017). By using APEX2-mediated proximity biotinylation, endogenous proteins on the OMM and ERM of living human fibroblasts were captured and identified. By mining OMM and ERM proteomic data, it is reported that the tail-anchored OMM protein synaptojanin-2 binding protein SYNJ2BP was observed richly in both OMM- and ERM-targeted APEX2. The overexpression of SYNJ2BP dramatically increases ER-mitochondria MCSs mediated by RRBP1, SYNJ2BP’s binding partner on the ER membrane.

To further advance the capabilities of APEX in protein-protein interactions and MCSs, split APEX2 was engineered. APEX2 was divided into N- and C-terminal fragments (at amino acid 201/202) for protein complementation assays (Xue et al., 2017). In addition, split APEX2 tool with more efficiency was engineered using directed evolution (Han et al., 2019). A total of 20 rounds of fluorescence activated cell sorting (FACS)-based selections from yeast displayed fragment libraries produced a 200-amino-acid N-terminal fragment (with 9 mutations relative to APEX2) called “AP” and a 50-amino-acid C-terminal fragment called “EX.” AP and EX fragments were each inactive on their own but were reconstituted to give peroxidase activity when driven together by MCS. By AP fused to TOM20^1–34^ for targeting OMM and EX fused to Cb5^100–134^ for targeting ERM, respectively, the split APEX enzyme activity was reconstituted and ER-mitochondria MCS was visualized at higher resolution by EM through the combination of the DAB staining in HEK293T cells (Figure 4C; Han et al., 2019). Expectably, split-BioID and split APEX technologies that combine reporter-fragment complementation and proximity-dependent biotinylation will be promising tools to map MCSs and identify new tethers.

## Considerations of Various Approaches for Studying MCSs

In living cells, individual organelles are highly dynamic, so MCSs show a highly dynamic spatiotemporal pattern to response to various external stimuli. For example, ER enable enlarge its cytoplasmic volume from ∼35 to 97%, and mitochondria from ∼10 to 70% in 15 min in COS-7 cells (Valm et al., 2017), which drives transient alteration of MCSs. As described in previous chapters, although several different types of microscopies and various biochemical techniques are available for the study of MCSs, their principal drawbacks should be considered to avoid to gain the false positive or false negative results.

**FIGURE 5:**
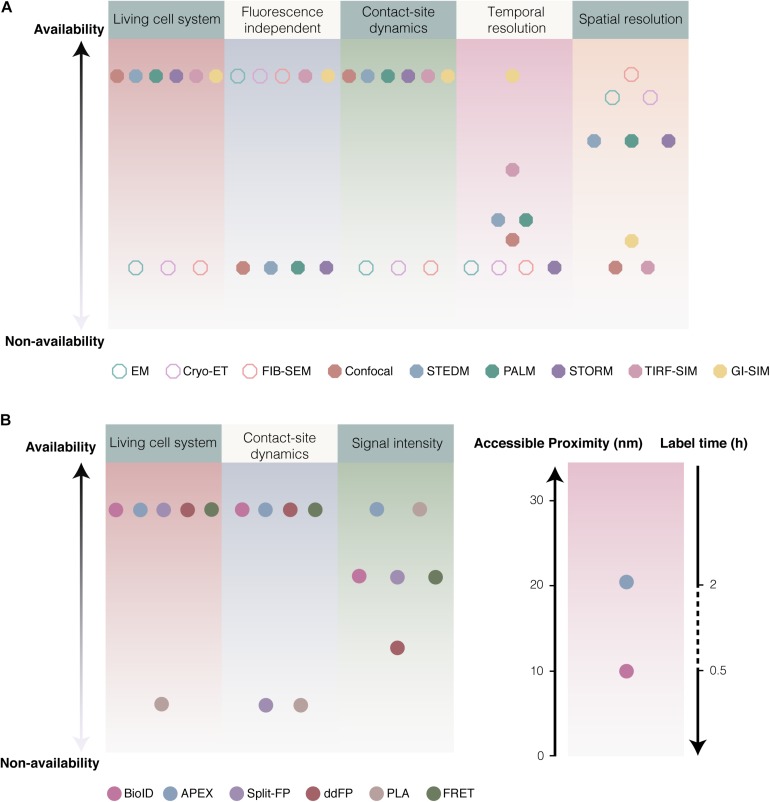
Considerations of various approaches for studying MCSs. **(A)** Comparison of various microscopies. Imaging of EM, Cryo-ET and FIB-SEM are fluorescence independent with high resolution (1.8–4 nm), rather than suitable for the dynamics of MCSs in living cell. Comparison to confocal (<0.5 Hz, ∼200 nm) (Schulz et al., 2013), super resolution fluorescence microscopy like STEDM (Schneider et al., 2015), PALM (Betzig et al., 2006; Gould et al., 2009) and STORM (<10 Hz, ∼20 nm) (Rust et al., 2006), GI-SIM (266 Hz and 97 nm) (Guo Y. et al., 2018) as well as combination TIRF with SIM (TIRF-SIM: ∼11 Hz, 100 nm) (Kner et al., 2009) showed extreme high temporal and spatial resolution. **(B)** The availability of various biochemical techniques. Generally, 12–24 h was required for biotinylation by BioID, while biotinylation based on APEX2 required ∼30 min. BirA* enable to effectively label proteins at distance ∼10 nm, and APEX at ∼20 nm.

For the electron and light microscopy, spatiotemporal resolution and imaging condition have been supposed to be crucial for the study of MCSs. EM, FIB-SEM, and cyro-ET of cell specimen at the nanometer scale enable observation of subcellular structures of MCSs without fluorescence labeling. Although EM and FIB-SEM provide the fine structure details of MCSs with extreme spatial resolution, the fixation and dehydration procedures of EM tend to disrupt the intact architecture of intracellular organelles and MCSs. Cyro-ET allows thin samples to be imaged in 3D in a nearly native state by immobilizing samples in non-crystalline ice without fixation and dehydration procedures. However, like EM and FIB-SEM, cyro-ET is impossible to detect the dynamics of MCSs in living cells (Figure 5A). Light microscopy based on fluorescence was developed in living cells to overcome fixation procedures and low through-put of EM. Confocal microscopy is one of the most common methods to visualize the subcellular localization of fluorescent membrane resident proteins, however, confocal microscopy alone is not good choice to detect membrane contacts because of low spatiotemporal resolution. The combination of confocal microscopy and biochemical techniques (proximity-driven fluorescent signal generation) confers to powerful approaches for the study of MCSs. Super-resolution fluorescence microscopy (SRM), such as STEDM, PALM, STORM, and SIM, offers a unique window with super temporal and spatial resolution in living cells for MCSs. Especially, GI-SIM show extremely great advantage in the study of MCSs dynamics because of the extreme high temporal and spatial resolution. Compare to EM, fluorescence labeling of organelles is requisite to light microscopy. In addition, SRM imaging are mostly available in imaging facilities and a small number of laboratories due to its high cost, even some of them are not commercial (Figure 5A).

The reversible biochemical tools are required to catch the dynamics of MCSs. PLA is able to detect protein-protein interactions on endogenous levels and does not require a superior quantity of protein in comparison to traditional fluorescent fusion protein expression. More importantly, the quantitative analysis of MCSs can be accessed by PLA. However, some antibodies for endogenous proteins are not available. The fixation step is also necessary for PLA, which renders PLA to monitor the dynamic MCSs. FRET between donor- and acceptor-tagged membrane proteins is a quite suitable tool for dynamics of MCSs because FRET is dependent on non-interacted and distance-dependent fluorescence excitation. The optimization of FRET sensor and special microscopy set-up, however, are required. Notably, the equivalent expression of donor and acceptor are required in cell for the study of MCSs. BiFC gives the bright fluorescence when driven together by inter-organelle membrane proximity and is a widely used biochemical tool for the study of MCSs. BiFC allows to detect the transient organelles contact. The drawbacks of BiFC should be carefully considered. Irreversible binding of split FP fragments leads to accumulation of the fluorescent signal and stabilizes the MCSs, which could cause false-positive signals (Eisenberg-Bord et al., 2016). In brief, based on reversible dimerization, ddFP is a highly effective indicator of inter-organelle membrane proximity. In ddFP, low-fluorescence intensity of the probes could restrict its application. Except for PLA, the approaches based on proximity-driven fluorescent signal generation enable identification of MCSs in high confidence and high dynamic state in living cells. Therefore, the above biochemical techniques are widely used to study MCSs and their principal drawbacks should be considered (Figure 5B).

The proximity-dependent biotinylation approaches have been supposed to a promising tool to identify new tethers involving in MCSs. Because of dynamics of MCSs, labeling time is a crucial parameter when the proximity-dependent biotinylation approaches are employed to study MCSs in living cells. Generally, 12–24 h was required for biotinylation by BioID, and it take at least 3 h to finish labeling (Youn et al., 2018; Gingras et al., 2019). Instead, the biotinylation based on APEX2 required ∼30 min only, that is an obvious advantage of APEX2 compared with BioID (Figure 5B). In addition, BirA^∗^ is able to biotinylate the potential proteins using exogenous biotin as well as endogenous biotin. Whereas, APEX2 catalyzes the substrate biotin-phenol that is only from external supplement. If BirA^∗^ is anchored to membrane contact by transfection the fusion construct, it is possible to capture false positive proteins because of long labeling time and utilization of endogenous biotin. A new technology called TurboID or miniTurbo was reported to finish biotinylation in 10 min in HEK293 cells (Branon et al., 2018; Mair et al., 2019).

Inter-organelle membrane contact sites allow subdomains of organelle membranes to contact within 10–50 nm, in which membranes were considered to be spatial proximity and even exist lipids and signal (Ca^2+^) transfer. For the biochemical techniques, it should be considered whether the distance reached by tools is suitable for the spatial proximity between inter-organelle membranes. PLA might lengthen its effective distance up to 30 nm (even more wider) as the adjustable length of nucleic acid arms and antibody affinity, which cause more false positive signals. FRET is able to detect a narrow contact with a typical range of roughly 1–10 nm (and up to 10 nm for atypical FRET pairs). Recently, split-GFP-based contact site sensor (SPLICS) was engineered to measure narrow (8–10 nm) and wide (40–50 nm) juxtapositions between ER and mitochondria by increasing the length of the spacer of the probe and resident protein. In addition, labeling radius of BioID/APEX should be considered, BirA^∗^ enable to effectively label proteins at distance ∼10 nm (Kim et al., 2014), and APEX labeling ∼20 nm (Gingras et al., 2019). Therefore, the linker length between probe/enzyme and resident protein should be seriously considered when the proximity-driven fluorescent signal generation and proximity-dependent biotinylation are employed to study MCSs.

Given the diameter of general subcellular apparatus, BioID and APEX enable to ensure the signal reliability to uncover potential tethers (Figure 3B). However, some tethers involving in wide contacts could be missed because labeling radius of BioID/APEX does not reach the distance between the enzyme and candidate tethers. If the linker length between bait and BirA^∗^/APEX is too long, it is easy to lead lots of false positives. To overcome this drawback, multiple baits or split-BirA^∗^/APEX could be used to map MCSs. The remodeling of AP and EX in split-APEX be thought to maintain normal MCSs (Han et al., 2019). Instead, EGFP fusion overexpression always cause abnormal membrane contact (Snapp et al., 2003; Hung et al., 2017).

Overall, the drawbacks and limitations should be considered to keep in mind when these approaches are used to the study of the dynamic MCSs.

## Conclusion and Perspectives

The various microscopies, proximity-driven fluorescent signal generation and proximity-dependent biotinylation have greatly accelerated the recent advances of MCSs at the molecular and system level. The drawbacks of various approaches should be considered to keep in mind when these approaches are used for the study of dynamic MCSs. To overcome the limitations, the approaches combination would be a better choice for the study of MCSs.

In the near future, full scenario of MCSs will be presented using the current and emerging approaches. On one hand, the high temporal-spatial resolution microscopies will be used to draw the extensive MCSs (organelles interactome). On the other hand, biochemical techniques, especially proximity-driven biotinylation coupled with MS-based proteomics will be sharpest tools to map MCSs and identify new tethers involving in MCSs, termed as “tether-omics.” In addition, cellular functions of MCSs and the role of MCSs in disease will be addressed.

## Author Contributions

All authors listed have made a substantial, direct and intellectual contribution to the work, and approved it for publication.

## Conflict of Interest

The authors declare that the research was conducted in the absence of any commercial or financial relationships that could be construed as a potential conflict of interest.
